# Point of Care strategy for rapid diagnosis of novel A/H1N1 influenza virus.

**DOI:** 10.1371/currents.RRN1039

**Published:** 2009-09-21

**Authors:** Antoine Nougairede, Laetitia Ninove, Christine Zandotti, Xavier De Lamballerie, Celine Gazin, Michel Drancourt, Bernard La Scola, Didier Raoult, Remi N. Charrel

**Affiliations:** ^*^Fédération de microbiologie, Assistance publique-hôpitaux de Marseille, Marseille, France ; Unité des virus Emergents, UMR 190 "Emergence des pathologies virales", Université de la Méditerranée & Institut de Recherche pour le Développement, Marseille, France.; ^‡^Fédération de microbiologie, Assistance publique-hôpitaux de Marseille, Marseille, France. and ^¶^Fédération de microbiologie, Assistance publique-hôpitaux de Marseille, Marseille, France ; Unité de Recherche sur les Maladies Infectieuses et Tropicales Emergentes UMR CNRS 6236 IRD 3R198, IFR 48, Faculté de Médecine, Université de la Méditerranée, Marseille, France.

## Abstract

In late June 2009, we implemented for public hospitals of Marseille Point Of Care strategy for rapid diagnosis of novel A/H1N1 influenza virus. During two months, we have tested more than 900 specimens in both Point Of Care laboratories. We believe that implementation of Point of Care strategy for the largest number of suspects cases may improve quality of patients care and our knowledge of the epidemiology of the pandemic.

## Introduction

        In late April 2009, The World Health Organization (WHO) announced the emergence of a novel A/H1N1 influenza virus. This virus spread rapidly, and after two months the WHO raised the alert level from phase 5 to phase 6 defining the first influenza pandemic of the 21^st^ century [1]. At the beginning of the pandemic, some countries established measures to identify all possible cases, but rapidly and due to the constant increase of suspected cases, they decided to stop the systematic screening.

         In France, the initial strategy started at the end of April 2009 was relying on the early identification of suspect cases which were directed into the hospital system to be tested for A/H1N1 virus, the positive cases being further hospitalized in isolation ward to prevent secondary transmission. This approach allowed a good knowledge of the kinetics of the pandemic when the large majority of positive cases are acquired abroad. The systematic screening of all suspect cases was stopped the July 7^th^ and replaced by sentinel systems to estimate the number of cases from surveillance of certain populations and to target groups with higher risk of morbidity or mortality [2]. The recent loss of laboratory-confirmed cases has rendered impossible to have now a reliable estimation of the evolution of the pandemic in France. Figures varying from 20,000 (Groupes Régionaux d'Observation de la Grippe, France) to 5,000 (Institut de veille sanitaire, France) cases weekly are claimed depending on the source data [3]. The data gathered at the beginning of the pandemic clearly indicate that the positive predictive value of the consultation by general practitioners or infectious diseases specialists is very low (15%). This is a strong argument to maintain laboratory confirmation of A/H1N1 suspect cases to provide solid epidemiological data. Even if there may be bias, the calculated proportion of laboratory-confirmed A/H1N1 cases amongst suspect cases allows to extrapolate the total number of cases occurring during the study period. Because of the innate evolutive nature of an epidemic/pandemic of transmissible disease, this extrapolation must be updated periodically from unambiguous data such as laboratory-confirmed diagnostics.

         Moreover to assess influenza morbidity and mortality, the approach based on systematic screening is more efficient than passive methods that produce under-estimate data [4]. Specifically, they cannot consider the role of unrecognized influenza infection as decisive co-morbidity factor in patients with underlying cardiovascular disease, hypertension, chronic pulmonary diseases and endocrine disorders. Therefore passive surveillance tends to hinder the knowledge of epidemiology of this pandemic [4]. Systematic detection for each patient with severe influenza-associated pathology like acute respiratory distress syndrome [5] or for each patient with high mortality risk like pregnant woman [6] may contribute to appreciate the incidence of influenza infection in this specifics groups.

         In this context, we believe that implementation of a rapid A/H1N1 influenza diagnostic for the largest number of suspects cases may improve the level of quality of patients care, the monitoring of pandemic through the reduction of secondary cases, and our knowledge of the epidemiology of the pandemic.

## Point of Care strategy and results

         In Marseille, the recent reorganization of the health structures (4 public hospitals) was achieved through a unique core laboratory for Clinical Microbiology. Centralization of laboratories such that they serve several hospitals allow to maximize the efficiency of testing at the lower cost but also have disadvantages like poor communication with clinical physicians and problems of specimens transport [7]. As countermeasures, we decided to build up two Point Of Care (POC) laboratories which are located in the vicinity of emergency units (see Figure 1) permit to shrink delays due to sample transport and to drastically reduce times for results for selected analyses. Open 24h/24 and operated by one person, POC laboratory can perform rapidly a large panel of analyses (see table 1). For selected parameters, the results were confirmed by the core laboratory.

**Figure:**
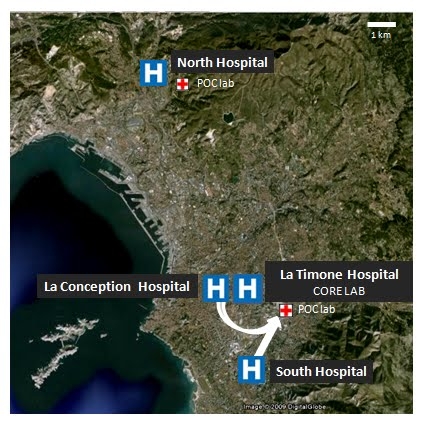
         Figure 1: POC laboratories organization in Marseille

**Figure:**
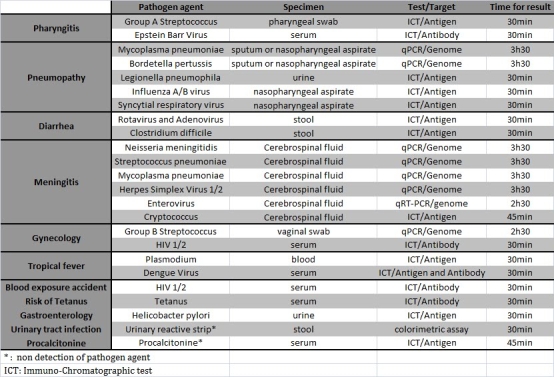
                                                                        Table 1: List of POC analysis.

        When secondary cases of influenza A/H1N1 were documented in France, we decided to implement rapid diagnosis of pandemic A/H1N1 virus in POC laboratory to investigate the level of virus circulation. Both POC laboratories were adapted to manage specimens processing in appropriate biosafety conditions, and to perform A/H1N1 virus detection via immuno-chromatographic tests (ICT) and one step qRT-PCR on SmartCycler (Cepheid). POC manipulators were trained to perform these analyses and to work with class II pathogen agents (see figure 2). For PCR testing we selected the qRT-PCR assay recommended by the French Influenza Reference National Center which was adapted to be run on SmartCycler. In parallel, all specimens were spiked with an internal control which allowed to monitor all steps through specific qRT-PCR detection. Comparative evaluation with the same protocol operated onto thermocycler MX3005P (stratagene) showed a lower sensitivity for low copy number specimens, but no significant difference in term of specificity. The core laboratory was processing all samples received at POC laboratories with (i) the qRT-PCR A/H1N1 assay aforementioned and (ii) a qRT-PCR SYBR Green system which detect all Influenza A viruses [8]. Both reactions were performed onto either Mx3005p thermocycler (Stratagene) or LC480 thermocycler (Roche). The maximal flow capacity (upper limit) of the POC laboratory for detection of A/H1N1 virus is 36 samples per day (see figure 2).

**Figure:**
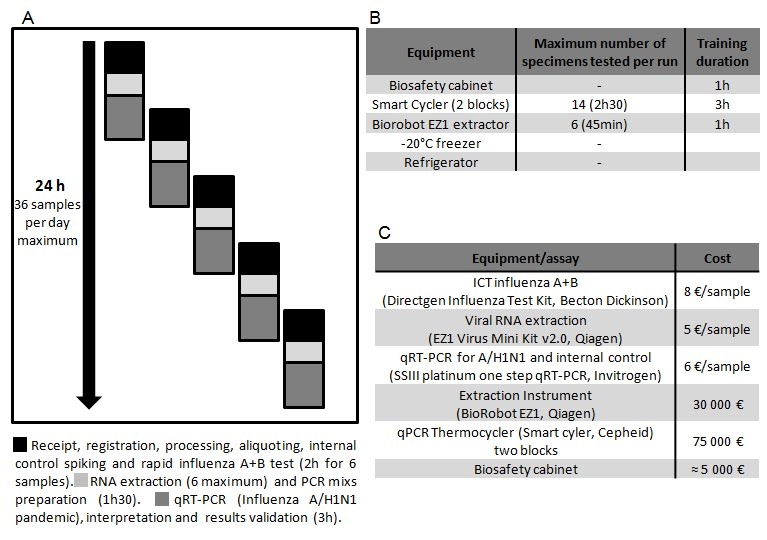
Figure 2: Flow capacity in POC lab for detection of Influenza A/H1N1 pandemic virus (**A**), and equipment/cost required for POC lab (**B,C**).

         From April 25^th^ 2009 to August 31^th^, the core laboratory received and processed 1815 samples to be tested for the presence influenza A/H1N1 virus. A total of 236 samples were positive (13%). During the entire period, our laboratory was in charge of testing human samples corresponding to 9 French departments (south-eastern France) representing 8 millions of inhabitants. From April 25^th^ and late June, the attitude recommended by the authorities was to test all suspect cases. During this period a maximum of 50 specimens were received weekly. On June 23^th^ 2009, we began to implement the POC strategy for all samples received from Marseille publics hospitals, while samples received from hospitals and practitioners outside of Marseille were processed directly in the core laboratory. This strategic change was synchronous with a drastic increase of weekly volume of samples. At this period, about 100-150 samples were received per week until late August which witnessed a novel obvious increase in the activity with 230 and 350 samples for the two latest weeks (see figure 3).

**Figure:**
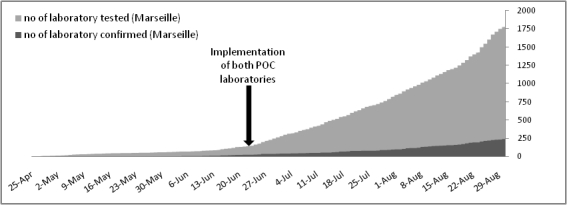

**Figure:**
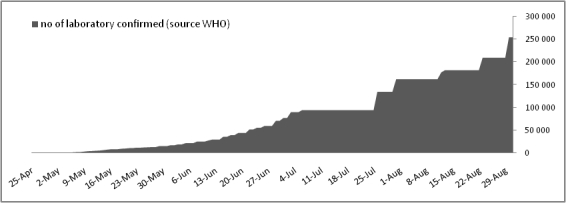
Figure 3: Cumulative number of laboratory confirmed cases of Influenza A/H1N1 pandemic infection announced by WHO [9](panel 1) and observed in Marseille virology laboratory (panel 2).

    From June 23^th^ 2009 to August 31^th^ 2009, 990 samples were analyzed in POC laboratories (see figure 4). A majority of samples originated from emergency wards (pediatrics and adults) (66.3%) and from the specific influenza consultation located in the North Hospital (19.2%). The respective activity of the two POC laboratories was similar. Each laboratory tested about 30-40 samples per week, but the last two weeks showed an increasing activity with respectively 149 and 191 samples tested (see figure 4). 

**Figure:**
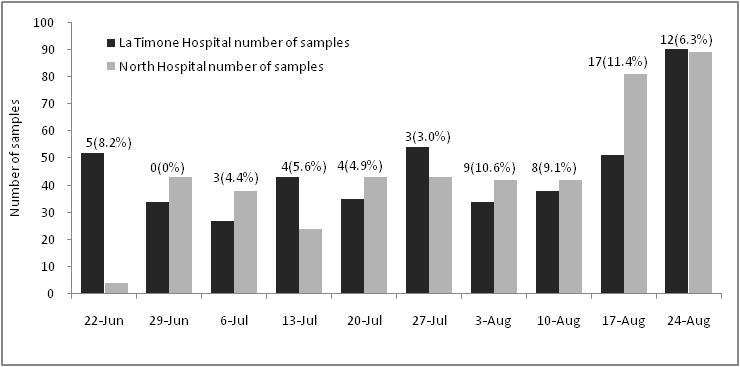
 Figure 4: Time distribution of samples tested for each POC. The number upon the bar chart represents the number of positive samples (% of positives samples).

    Among the 990 samples tested in POC laboratories, 21 were positive with rapid ICT. After RNA extraction, some samples were tested directly by the core laboratory. A total of 563 samples were tested using the one step qRT-PCR on SmartCycler, of which 33 (5.9%) were positive. Finally, 65 samples were positives after core laboratory confirmation test (6.6%). Therefore, the sensitivity of POC strategy for A/H1N1 was 63,1% after rapid ICT and 84.6% after qRT-PCR assay. We determined the positive and negative predictive value (PPV and NPV) for each step of POC process (see figure 5).

**Figure:**
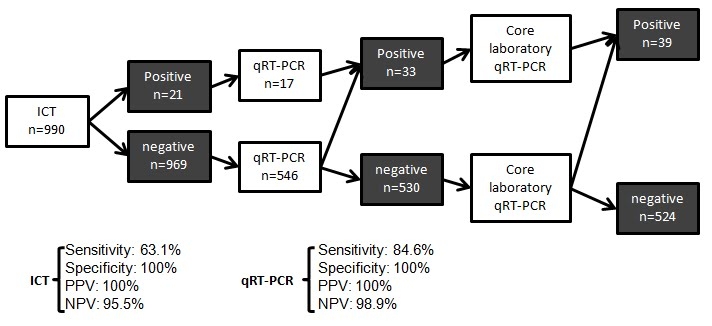
 Figure 5: Flow chart of POC samples with sensitivity, specificity, PPV and NPV of each step.

         The number of positive samples fluctuated between weeks but increased during the last two weeks. Percentages also fluctuated but increased during the last four weeks with positive results representing more than 10% of tested specimens.

         The time necessary to obtain the results was 1-2h for the 21 samples positives with rapid ICT and 4-7h for the others. Computer-based communication of results allowed to save time dedicated to telephone calls for both physician and POC person.

## Discussion

         POC strategy for A/H1N1 requires minimal training (8 hours with written protocols), necessitates a small dedicated laboratory area which can be implemented in any hospital settings, and can be operated by one person at the time. It allows diagnosis within the clinical time that can help the decision to treat the patient, to isolate the patient or to discharge the patient. It provides reliable data to timely study the local epidemiology and its evolution. It helps to define and to monitor the epidemiology at a larger scale (regional, national, international) via extrapolation based on the percentage of confirmed cases over suspect cases.

         The usefulness of POC strategy in the context of A/H1N1 pandemic proves that POC should be implemented throughout the country not only for emergency situations but also as a daily tool to improve the quality of care for hospitalized patients via shortening the delays and allowing decisions within the clinical time. It is likely that hospitalization costs will also directly beneficiate from POC strategy, through reduction of the duration of hospitalization as previously demonstrated [10] [11].

         The decision to abandon systematic laboratory testing of suspect patients has been equivalent to break the thermometer and to attempt to define body temperature curve. We believe that implementing diagnostic tools for pulmonary infections including flu may allow to fight efficiently this group of diseases that is the more common cause of death worldwide [12] and the most neglected cause of life reduction [13]. In the current situation where less than 10% of tested specimens are found positive, the economic impact of rapid testing through the POC strategy is very important. Indeed, all negative patients - the large majority of suspect patients - can therefore go back to their professional activities immediately. Beside, readmission of suspect students in the educational course is often conditioned by a certificate assessing the absence of contagiousness, which need to be based on specific virological diagnostic such as implemented in the POC strategy.

## Funding information

No specific funding was received for the work presented in this manuscript.

## Competing interests

The authors have declared that no competing interests exist.
